# The need for uniform and coordinated practices involving centrally manufactured cell therapies

**DOI:** 10.1186/s12967-022-03385-9

**Published:** 2022-04-25

**Authors:** David Stroncek, Anh Dinh, Herleen Rai, Nan Zhang, Rob Somerville, Sandhya Panch

**Affiliations:** 1 Center for Cellar Engineering, Department of Transfusion Medicine, NIH Clinical Center, Bethesda, MD USA; 2 Center for Cellular Engineering, Department of Transfusion Medicine, NIH Clinical Center, 10 Center Drive, MSC–1184, Building 10, Room 1C711, Bethesda, MD 20892-1184 USA

**Keywords:** Cellular therapies, Cancer immunotherapies, Gene therapies

## Abstract

Cellular therapies have become an important part of clinical care. The treatment of patients with cell therapies often involves the collection of autologous cells at the medical center treating the patient, the shipment of these cells to a centralized manufacturing site, and the return of the cryopreserved clinical cell therapy to the medical center treating the patient for storage until infusion. As this activity grows, cell processing laboratories at many academic medical centers are involved with many different autologous products manufactured by several different centralized laboratories. The handling of these products by medical center-based cell therapy laboratories is complicated and resource-intensive since each centralized manufacturing laboratory has unique methods for labeling, storing, shipping, receiving, thawing, and infusing the cells. The field would benefit from the development of more uniform practices. The development of a coordinating center similar to those established to facilitate the collection, shipping, and transplantation of hematopoietic stem cells from unrelated donors would also be beneficial. In summary, the wide range of practices involved with labeling, shipping, freezing, thawing, and infusing centrally manufactured autologous cellular therapies lack efficiency and consistency and puts patients at risk. More uniform practices are needed.

## Commentary

Cellular therapies have become an important part of clinical care. Chimeric Antigen Receptor (CAR) T-cells are frequently used to treat acute lymphocytic leukemia [1], lymphomas [2] and multiple myeloma [3]. T-Cell Receptor engineered T-cells directed to Human Papilloma Virus (HPV) oncoprotein E7 have shown promising results for the treatment of HPV-associated cancers [4]. As a result of this success, many cellular therapies are in clinical trials, and some have been licensed. While phase I and II cell therapy clinical trials are typically performed at a single center, the treatment of patients on later phase clinical trials and with licensed products involves multiple treatment centers. Since many of these cell therapies are made from a patient’s own cells or are autologous therapies, treatment of patients on multicenter trials typically involves manufacturing at a centralized laboratory.

The manufacturing of autologous cancer cellular therapies typically involves the collection of the cell population of interest by apheresis or by simple phlebotomy at a center where the patient is being treated. Collected cells are sent to the medical center’s cell processing lab, where the containers holding the cells are labeled and then shipped to a centralized laboratory to manufacture the cellular therapy. The medical center-based cell processing laboratory may immediately ship the cells to the centralized laboratory without manipulating or processing them, or cryopreserve and store the cells until shipment.

After the centralized laboratory receives the cells, the autologous cell therapy is manufactured over several days. Since products from multiple patients may be manufactured concurrently, care must be taken by the centralized laboratory to maintain unique identifiers on the cells throughout the manufacturing process in order to prevent patient product mix ups. After the manufacturing process is completed, the cell therapy is cryopreserved and stored. Lot-release testing is then completed, and the cell therapy is shipped to the medical center cell processing laboratory where the patient is being treated. The medical center cell processing laboratory receives, stores, thaws, and issues the cells for the infusion in the clinic or patient care unit.

While the most complicated and resource-intensive activity in this process is the centralized manufacturing of the cells, the activities of the medical center-based cell processing laboratory require a considerable amount of work. Our medical center-based cell processing laboratory first became involved with these activities more than 10 years ago. This activity continues to grow, and we are now involved with several unique autologous products manufactured by a variety of biotech laboratories. We have found that each centralized manufacturing laboratory has specific labeling, storage, shipping, receiving, thawing, and infusion requirements. This variability creates additional work for medical center-based cell processing laboratory staff. In addition, our and other medical center-based laboratories have dedicated staff who spend a significant amount of time managing logistics with each centralized manufacturing laboratory, including the coordination of product shipping and receipt [5].

Handling these autologous products is not only resource-intensive and cumbersome for the medical center-based cell processing laboratory, but it creates potential patient safety issues. For many of these cell therapies, our laboratory handles only 2 to 3 products annually for each clinical protocol, making it difficult for staff to stay proficient. In addition, the companies responsible for manufacturing the cellular therapies often change their procedures. The low level of activity and frequent modifications increase the chances of errors related to product labeling or thaw and infusion that could endanger the patient or result in product discard/wastage.

The field would benefit from the development of more uniform practices. Protecting the intellectual property involved with a biotech’s manufacturing process is important, but this should not impact labeling and shipping of the starting cellular material and receiving, thawing, and infusing the clinical cellular therapy. While standards and conventions have been developed for labeling of cell therapies, there is wide variation in the methods used for cryopreservation and thawing cells. Cell processing laboratories have been cryopreserving and thawing cellular therapies for many years using several different methods. The methods traditionally used came from research laboratories and are highly variable. More recently, a number of instruments, supplies, and reagents designed specifically for cryopreserving, storing and thawing cells in a Good Manufacturing Practices (GMP) setting have become available. While the wide variety of cell types and qualities of cells being used clinically does not allow for the use of a single method, the development and use of best cryopreservation and thawing practices using GMP reagents are needed. Some standardization of the containers used to store the cell therapy, as well as cell infusion sets, would also be worthwhile.

The current situation is similar to the field of unrelated donor transplantation in the 1980s. At that time, blood centers identified people who donated platelets for transfusion, who had been Human Leukocyte Antigen (HLA) typed, and would agree to donate marrow to an unrelated individual. A number of blood centers developed these registries of unrelated marrow donors, which allowed marrow transplants to occur among HLA-compatible unrelated donors and recipients and become widely available. However, searching for donors and coordinating the collection and transportation of marrow from the marrow collection center to the transplant center was labor-intensive. Each transplant center had to be in contact with multiple blood centers to identify a donor, and once a donor was identified, each blood center had its own policies and practices for collecting and shipping marrow. These policies and practices often differed among centers. Consequently, the US government provided funds to establish the National Marrow Donor Program (NMDP), which maintained a single donor registry [6, 7]. In addition, the NMDP developed uniform practices and coordinated the collection and shipment of marrow to transplant centers. While the production of autologous cell therapies does not require searching for HLA-compatible donors, the field would likely benefit from an organization similar to the NMDP to create uniform policies and practices and coordinate the collection and shipment of the starting cellular material and the shipment of the final cellular therapy.

In summary, the current wide range of practices involved with labeling, shipping, freezing, thawing, and infusing centrally manufactured autologous cellular therapies creates unnecessary complexities and places patients at risk. More uniform practices are need. Toward this goal, an organization to coordinate the scheduling, shipment, and receipt of the cellular material used to manufacture the cells and the final cell therapy would be beneficial.

## Data Availability

Not applicable.

## Abbreviations

CAR: Chimeric antigen receptor
HPV: Human papilloma virus
HLA: Human leukocyte antigen
GMP: Good manufacturing practices
NMDP: National marrow donor program
